# Left Atrial Deformation in Paediatric Dilated and Hypertrophic Cardiomyopathy: Insights from Two-Dimensional Speckle-Tracking Echocardiography

**DOI:** 10.3390/jcm14248622

**Published:** 2025-12-05

**Authors:** Iolanda Muntean, Beatrix-Julia Hack, Diana-Ramona Iurian, Theodora Benedek, Diana Muntean, Ioana-Octavia Matacuta-Bogdan, Asmaa Carla Hagau

**Affiliations:** 1 Department of Paediatrics III, George Emil Palade University of Medicine, Pharmacy, Science, and Technology of Târgu Mureș, 540136 Targu Mures, Romania; iolanda.muntean@umfst.ro; 2 Clinic of Pediatric Cardiology, Emergency Institute for Cardiovascular Diseases and Transplantation of Targu Mureș, 540139 Targu Mures, Romania; dianaiurian@yahoo.com; 3 Doctoral School of Medicine and Pharmacy, George Emil Palade University of Medicine, Pharmacy, Science, and Technology of Targu Mures, 540136 Targu Mures, Romania; asmaa.carla@yahoo.com; 4 6th Medical Department, George Emil Palade University of Medicine, Pharmacy, Science, and Technology of Târgu Mureș, 540136 Targu Mures, Romania; theodora.benedek@gmail.com; 5 Department of Cardiology, County Emergency Clinical Hospital, 540139 Targu Mures, Romania; 6 Faculty of Medicine, George Emil Palade University of Medicine, Pharmacy, Science, and Technology of Targu Mures, 540136 Targu Mures, Romania; muntean.diana.24@stud.umfst.ro; 7 Faculty of Medicine, Lucian Blaga University, 550106 Sibiu, Romania; ioana.matacutabogdan@ulbs.ro; 8 Pediatric Clinical Hospital, 550106 Sibiu, Romania

**Keywords:** atrial strain, speckle-tracking echocardiography, pediatric heart failure, pediatric cardiomyopathy

## Abstract

**Background**: Left atrial strain (LAS) derived from speckle-tracking echocardiography (STE) provides a sensitive, load-dependent measure of atrial function and ventricular filling pressures. Data on LAS in paediatric cardiomyopathies are still scarce; therefore, this study aimed to assess LA phasic function in dilated (DCM) and hypertrophic (HCM) cardiomyopathy and to determine its relationship with clinical and echocardiographic indices of disease severity. **Methods**: We conducted a cross-sectional case–control study that included 84 children (DCM n = 29, HCM n = 29, control n = 26) who underwent comprehensive clinical and echocardiography evaluation, including LAS parameters (reservoir—LASr; conduit—LAScd; and contractile—LASct). Group comparisons were performed using ANOVA or Kruskal–Wallis tests with post hoc adjustments, and correlations were analysed using Pearson’s or Spearman’s coefficients. Multivariable linear and logistic regression models were adjusted for age, body surface area (BSA), heart rate (HR), and blood pressure (BP) percentiles. **Results**: LASr and LAScd were significantly reduced in both cardiomyopathy groups compared with controls (*p* < 0.001), following a graded pattern (DCM < HCM < control). In DCM, lower LASr was independently associated with higher left atrial volume index (LAVi) and elevated E/E′ ratio, whereas in HCM, septal hypertrophy (IVSd Z-score) and log NT-proBNP were dominant determinants of impaired LASr. In logistic regression, LASr (OR = 0.93, *p* = 0.016) and LAScd (OR = 1.21, *p* = 0.001) independently predicted severe NYHA/Ross functional class after covariate adjustment, while LASct showed no significant association. **Conclusions**: These findings demonstrate that LA reservoir and conduit strain are markedly impaired in paediatric cardiomyopathy and are strongly linked to structural remodelling and functional limitation, underscoring their value as sensitive non-invasive markers of disease severity.

## 1. Introduction

Dilated and hypertrophic cardiomyopathies represent the most common primary cardiomyopathies in children [1]. Although they have a low incidence of approximately 1.1–1.5 per 100,000 individuals under 18 years of age, they carry substantial morbidity and mortality, partly due to the scarcity of paediatric data and the frequent need to extrapolate diagnostic and management strategies from adult studies [2].

Diastolic impairment represents a common characteristic finding in both dilated (DCM) and hypertrophic (HCM) cardiomyopathy, although the underlying mechanisms differ between the cardiomyopathies. In HCM, impaired relaxation and increased myocardial stiffness lead to elevated left ventricular (LV) filling pressures, resulting in progressive left atrial (LA) dilatation, increased wall stress, and mechanical dysfunction [3]. Conversely, in DCM, the combination of systolic impairment and LV dilatation creates a chronic volume and pressure overload on the LA, resulting in compensatory remodelling and reduced reservoir, conduit, and pump function [4]. In both cases, the alterations in the LA mechanics reflect chronic exposure to increased LV filling pressures and serve as an indirect indicator of diastolic dysfunction and disease progression.

The physiologically normal LA exhibits three distinct phasic roles across the cardiac cycle, each phase contributing to optimal LV filling and cardiac performance. First, during the ventricular systole and isovolumic relaxation, the LA acts as a reservoir, collecting pulmonary venous return while storing elastic energy. Second, when the mitral valve opens in early diastole, the LA functions as a conduit, allowing the passive flow from the pulmonary veins to the LV. Finally, in late diastole, atrial contraction provides a booster pump effect that increases the LV stroke volume by almost 20% [5]. These phasic elements—reservoir, conduit and contractile—are fundamental for diastolic function.

The assessment of LA function and morphology can be performed using various imaging modalities, including invasive techniques such as cardiac catheterisation and non-invasive techniques such as echocardiography, computed tomography, or magnetic resonance [6]. Among these, cardiac magnetic resonance (MRI) and computed tomography have demonstrated excellent accuracy and reproducibility in quantifying LA strain (LAS) due to superior spatial resolution and low interobserver variability [7]. However, their clinical use is often limited by high cost, increased acquisition protocols, and contraindications in certain paediatric or critically ill patients [8,9]. Therefore, echocardiography remains the first-line imaging modality for the non-invasive evaluation of LA function and LV diastolic dysfunction in clinical practice. In adult patients, there are established conventional indices that enable estimation of LV filling pressures and grading of diastolic function. For example, the American Society of Echocardiography (ASE) has outlined standardised LA volume assessment approaches, thereby helping reduce variability and improve clinical applicability [10]. Furthermore, within echocardiography, STE has emerged as a reproducible tool to quantify LAS, reflecting the dynamic LA deformation during each phase [7]. In adults, LAS has demonstrated strong correlations with invasive filling pressures, LA volume, and clinical outcomes in different cardiovascular diseases, such as heart failure (HF), HCM, or atrial fibrillation [11,12]. Despite its dependence on image quality and vendor-specific software, STE offers superior reproducibility compared to conventional Doppler parameters, and it is now regarded as the reference non-invasive method for LA functional analysis [6].

In contrast, diagnosing and grading diastolic dysfunction in the paediatric population remains challenging, as current guidelines are largely derived from adult data. Also, conventional echocardiographic parameters and tissue Doppler-derived indices (TDIs) are often difficult to apply in children. Moreover, studies have shown that these indices offer some discriminatory value, but their wide variability and frequent inconsistencies across individual patients limit their reliability [13]. Furthermore, substantial heterogeneity in paediatric echocardiography practice and training across Europe further underscores the need for standardised protocols [14]. Consequently, relying solely on conventional measurements may underestimate the presence or severity of diastolic abnormalities in paediatric cardiomyopathy. Also, although volumetric measurements at distinct phases of the cardiac cycle can evaluate LA function, it may be time-consuming and constrained by limited temporal resolution, especially at elevated heart rates (HRs). In this context, STE may provide a more direct assessment of atrial deformation and diastolic mechanics, with a faster and predominantly automated assessment method, potentially overcoming the limitations of traditional parameters used to characterise diastolic dysfunction in children [15].

Considering these factors, the current study aimed to evaluate LA phasic strain components—reservoir (LASr), conduit (LAScd), and contractile (LASct)—in paediatric patients with HF and DCM/HCM, compared with age-matched healthy controls. We also sought to investigate the relationships between LAS parameters, clinical severity, conventional echocardiographic indices, and biomarkers. By combining conventional methods with STE analysis, this study could offer insights into the determinants and clinical importance of LA dysfunction in paediatric cardiomyopathy.

## 2. Materials and Methods

### 2.1. Study Design and Ethical Approval

This study was designed as a prospective, cross-sectional case–control study and was conducted at the Clinic of Paediatric Cardiology of the Emergency Institute of Cardiovascular Diseases and Transplantation in Targu Mures, Romania. Data collection took place between January 2022 to December 2023. The study protocol received approval from the institutional Ethics Committee (approval number 1596/2022) and was carried out in accordance with the principles of the Declaration of Helsinki. Before entering the study, all participants and their parents or legal guardians received detailed information about the study objectives and procedures. Written informed consent was obtained for both participation and the anonymised use of clinical imaging data for scientific reporting.

### 2.2. Study Population

The study population comprised 84 paediatric patients divided into three groups: 29 patients diagnosed with idiopathic DCM (DCM group), 29 patients diagnosed with primary HCM (HCM group), and 26 age- and sex-matched healthy controls (control group). Inclusion criteria for the cardiomyopathy groups were confirmed diagnosis of idiopathic DCM or primary HCM based on the European Society of Cardiology guideline definitions, age between 1 month and 18 years, and complete feasibility of both conventional echocardiography and 2D-STE acquisition [16]. Diagnosis of cardiomyopathy was established using conventional echocardiographic criteria and supported by clinical findings. Dilated cardiomyopathy was defined by an increased LV end-diastolic diameter (LVEDD) ≥ 2 standard deviations (SDs) and global or regional systolic dysfunction, while HCM was characterised by LV wall thickening diameter ≥ 2 SDs. Exclusion criteria included congenital heart diseases, coronary or valvular abnormalities, systemic hypertension, neuromuscular or metabolic disorders, and systemic or infectious causes of cardiomyopathy. All consecutive patients meeting the diagnostic criteria during the study period were included, and 2D-STE analysis was feasible in all cases. Because no patients were excluded due to suboptimal image quality or incomplete data, a flowchart summarising patient selection was not required.

Healthy controls were recruited from children referred for benign cardiac screening (for example, innocent murmur or sports clearance). All controls had a normal clinical examination, normal electrocardiogram, and normal echocardiographic findings. Furthermore, no children had a history of cardiovascular, metabolic, systemic, or chronic disease. The matching with the cardiomyopathy groups was performed by frequency matching for age and sex. Also, the comparability of anthropometric parameters across groups was confirmed statistically to minimise selection bias.

### 2.3. Working Method

#### 2.3.1. Clinical Evaluation and Laboratory Assessment

All participants underwent a standardised clinical evaluation conducted by a paediatric cardiologist, including assessment of vital parameters such as heart rate (HR) and blood pressure (BP), as well as anthropometric indices, all corrected for age and size, according to CDC z-scores [17,18]. Heart failure severity was evaluated using age-appropriate paediatric classification systems: the Ross scale for children younger than 5 years and the New York Heart Association (NYHA) functional classification for older children and adolescents. For statistical purposes, both were standardised into two categories: mild (Class I–II) and severe (Class III–IV) [19,20].

On the same day as the clinical evaluation and echocardiography, venous blood samples were collected from all participants. Serum N-terminal pro-B-type natriuretic peptide (NT-proBNP) concentrations were determined using a chemiluminescent immunoassay on a fully automated analyser (Roche Cobas, Mannheim, Germany), following standard laboratory protocols. Age- and sex-adjusted z-scores for NT-proBNP were calculated using the validated online tool provided by the German Heart Centre Munich, and log-transformed values (zlog NT-proBNP) were used for statistical analysis [21,22].

#### 2.3.2. Echocardiography

Transthoracic echocardiography was performed using a Philips EPIQ 7 ultrasound system (Philips Medical Systems, Andover, MA, USA) equipped with multi-frequency transducers (5–12 MHz). All acquisitions and measurements followed the current recommendations of the ASE and the EACVI for paediatric echocardiographic assessment [23]. None of the participants required sedation.

Conventional echocardiographic measurements were obtained from standard parasternal and apical views. Parameters of LV systolic function included mitral annular plane systolic excursion (MAPSE), LV ejection fraction (LVEF), and LV fractional shortening (LVFS). Diastolic inflow velocities were measured using pulsed-wave Doppler at the tips of the leaflets to record early (E) and late (A) diastolic peak velocities, from which the E/A ratio was calculated. Additionally, measurements of LV were recorded, including interventricular septal thickness in systole and diastole (IVSs, IVSd), as well as left ventricular end-diastolic and end-systolic diameters (LVEDD, LVESD). Linear dimensions were standardised as z-scores based on the established paediatric reference values published by the Boston group [24,25].

Tissue Doppler imaging was performed in the apical four-chamber (A4C) view, at the level of the lateral mitral annulus. From the resulting spectral tracings, peak systolic velocity (S′), early diastolic velocity (e′), and late diastolic velocity (a′) were measured. The E/e′ and E`/A` ratios were calculated to estimate LV filling pressures. All TDI recordings were obtained with the ultrasound beam aligned as parallel as possible to the direction of myocardial motion to minimise angle dependency. Each parameter was averaged over three consecutive cardiac cycles to reduce beat-to-beat variability.

#### 2.3.3. Strain Analysis

Assessment of LA and LV deformations was performed using 2D STE. All analyses were performed offline using QLAB Cardiac Analysis software version 10 (Philips Healthcare, Andover, MA, USA) equipped with the TomTec Autostrain module, which enables semi-automated endocardial border detection and strain tracking across standard apical views.

Two-dimensional images were obtained from standard apical views (A4C, A3C and A2C) at frame rates ranging from 60 to 90 frames per second. Although higher frame rates may be beneficial in younger children with rapid HR, previous studies have demonstrated optimal reproducibility for LAS and LV global longitudinal strain (LV GLS) within this range, supporting its use in paediatric 2D-STE [26]. Sector width and depth were adjusted to maximise temporal resolution while ensuring complete visualisation of the myocardial borders. All cine sequences were saved in Digital Imaging and Communications in Medicine format for offline analysis.

**Figure 1:**
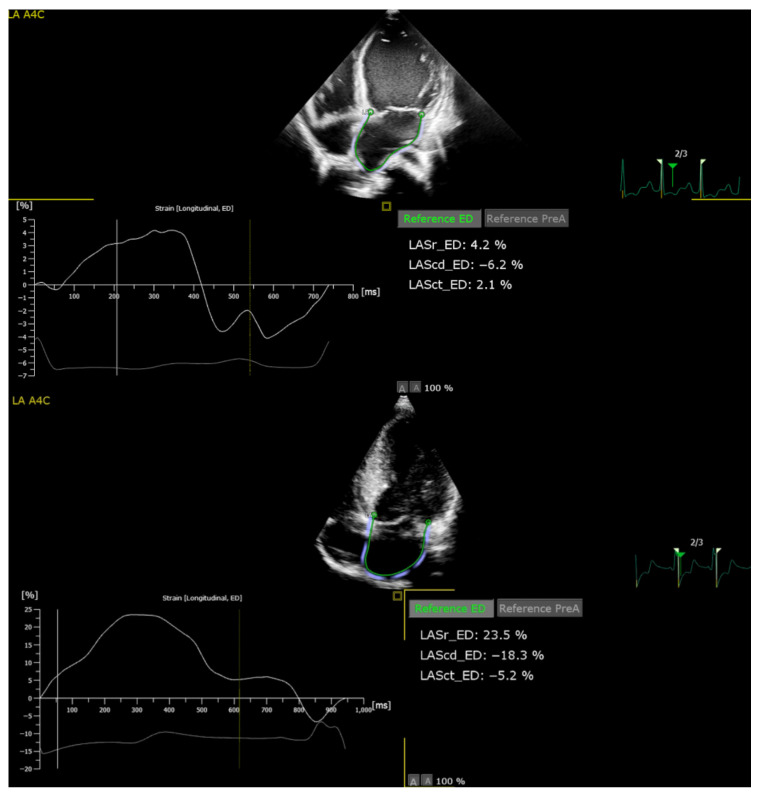
Apical four-chamber views illustrating LA phasic strain measurement using two-dimensional speckle-tracking echocardiography 2D-STE. The endocardial border was manually traced and automatically tracked throughout the cardiac cycle, generating longitudinal strain curves for the reservoir (LASr), conduit (LAScd), and contractile (LASct) phases. The upper panel shows an example from a patient with DCM, showing reduced deformation with LASr = 4.2%, LAScd = −6.2%, and LASct = −2.1%. The lower panel is an example from a patient with HCM, demonstrating markedly impaired phasic strain values (LASr = 23.5%, LAScd = −18.3%, LASct = −5.2%). Strain values were referenced to end-diastole (ED) and synchronised with the electrocardiographic signal for temporal alignment.

For each participant, three consecutive cardiac cycles were selected automatically by the software. After placing three key points on the LV endocardial border (basal septal, lateral and apical), the endocardial contour was generated automatically and subsequently adjusted manually to ensure accurate tracking and correct any artefacts. The LV GLS was calculated as the average peak systolic strain values derived from all segments across the three apical views, based on the 18-segment LV model implemented in QLAB 10. For LAS analysis, the endocardial border was manually traced in A4C views, excluding the pulmonary vein ostia and the LA appendage, in accordance with the latest ASE/EACVI consensus recommendations. The zero-strain reference was set at LV end-diastole (ED-gated), as recommended [27]. Using this convention, LASr was defined as the positive strain increment from ventricular ED to the peak of LA filling during LV systole; LAScd as the negative change from mitral valve opening to the onset of atrial contraction; and LASct as the negative change from the start of atrial contraction to the next ED-frame (Figure 1). Additionally, left atrial volume (LAV) was measured at end-systole and indexed to body surface area (LAVi, mL/m^2^) using the biplane area–length method. Measurements were averaged over three sinus beats, and acquisitions with poor tracking in more than two segments per view were excluded.

### 2.4. Statistical Tests Used

The statistical analysis was performed using IBM SPSS Statistics version 13 (IBM Corp., Armonk, NY, USA) and GraphPad Version 10.0 (GraphPad Software, San Diego, CA, USA). A two-tailed *p*-value < 0.05 was considered statistically significant.

Data distribution was evaluated using the Shapiro–Wilk test. Continuous variables were presented as median [interquartile range] or mean ± SD, as appropriate. Group comparisons were performed using one-way ANOVA or the Kruskal–Wallis test, as appropriate. Post hoc analyses were conducted using Tukey correction for normally distributed variables with homogeneous variances, Bonferroni correction for parametric comparisons where variance assumptions were not met, and Dunn–Holm post hoc correction for nonparametric pairwise testing. Categorical variables were summarised as absolute frequencies and percentages and were compared using the χ^2^ test or Fisher’s exact test. Associations between continuous variables were examined using Pearson or Spearman correlation analysis, depending on data distribution. Given the modest cohort size, all multivariable models were intentionally kept parsimonious. For logistic regression, each model included only one LAS parameter together with age, BSA, HR, and cardiomyopathy subtype (DCM or HCM) to minimise the risk of overfitting. Predictors were selected based on significant or borderline univariable association and clinical relevance, and the number of variables per model was restricted. Regression coefficients (b), standardised beta coefficients (β), 95% confidence intervals (CIs), and corresponding *p*-values were reported. Model performance was assessed by the coefficient of determination (R^2^ and adjusted R^2^) and analysis of variance (ANOVA) for overall model significance. Collinearity diagnostics (tolerance and variance inflation factor) were examined to exclude multicollinearity among predictors.

To assess the predictive value of LAS components for functional status, binary logistic regression analyses were performed with NYHA/Ross class dichotomised as mild (I–II) versus severe (III–IV) as the dependent variable. Independent variables included LAS parameters (LASr, LAScd, and LASct), each entered in separate models, adjusted for age, BSA, heart rate (HR), and cardiomyopathy subtype (DCM or HCM). Model fit was evaluated using the chi-square test for overall significance, Nagelkerke R^2^ for explained variance, and the Hosmer–Lemeshow test for calibration. Odds ratios (ORs) with 95% confidence intervals (CIs) were reported. A sensitivity analysis using Firth penalised logistic regression was additionally performed to assess model robustness in the context of a limited number of events.

## 3. Results

### 3.1. Baseline Characteristics

Among 84 participants (DCM n = 29, HCM n = 29, controls n = 26), sex distribution did not differ across groups (male: 72.4% in the DCM group, 62.1% in the HCM group, and 50.0% in the controls). Also, continuous variables were similar between groups.

Office systolic blood pressure (sBP) and sBP percentiles did not differ significantly among DCM, HCM, and controls. The numerically lower sBP in DCM probably reflects disease-related low output and vasoactive therapy, while lower percentiles in HCM may be affected by β-blockade.

Baseline characteristics of the patients are displayed in Table 1.

### 3.2. Cardiomyopathy Cohorts

The NYHA/ROSS distribution did not show a significant global difference between DCM and HCM (*p* = 0.07). However, there was evidence of a trend toward milder classes in HCM (*p* = 0.0457).

Ventricular arrhythmias were more frequent in the DCM than HCM (ventricular premature beats in 55.2% vs. 31.0%; ventricular tachycardia in 27.3% vs. 6.9%), but differences were not statistically significant. Atrial arrhythmias were uncommon in both groups (10.3% vs. 6.9%) and consisted only of premature atrial contractions and a single case of atrial tachycardia; no patient had atrial fibrillation or atrial flutter.

Beta-blocker use was similar between groups (approximately 85% of patients), whereas diuretics, ACE inhibitors and inotropes were almost exclusively used for DCM patients (all *p* < 0.01). Calcium-channel blockers were used in only one patient from the HCM group.

Z-log NT-proBNP levels were highest in DCM (3.28 ± 2.12), intermediate in HCM (1.99 ± 1.99), and lowest in controls (0.31 ± 0.64), showing the pattern DCM > HCM > Control (*p* < 0.01). All comparisons are shown in Table 1.

### 3.3. Echocardiography Parameters

*In conventional Doppler indices*, transmitral E velocity was comparable across groups (*p* = 0.57). The A-wave was higher in HCM versus controls (*p* < 0.01), with DCM intermediate and not different from either group after correction. The E/A ratio was lower in HCM than in controls (*p* < 0.01), while DCM again remained intermediate.

*Tissue Doppler* showed a clear disease signal: lateral e′ was reduced in both DCM and HCM versus controls (*p* < 0.01), with a borderline HCM–DCM difference (*p* = 0.056). Consistently, E/e′ was higher in DCM and HCM than in controls (*p* <0.01), and trended higher in HCM than in DCM (*p* = 0.078).

*Systolic function* showed the expected gradient: MAPSE increased stepwise from DCM to HCM to controls (*p* < 0.01; all Tukey pairwise significant), and lateral S′ was lower in both cardiomyopathy groups versus controls (*p* < 0.01) without a difference between DCM and HCM (*p* = 0.74). LV EF and LV SF differed across groups (*p* < 0.01). DCM showed markedly reduced LV EF and LV SF versus HCM and controls (all *p* < 0.01), while HCM and controls were similar (LV EF *p* = 0.40; LV SF *p* = 0.16).

*Left ventricle measurements*: IVS and IVS systolic Z-score differed across groups (both *p* < 0.0001). As expected, the HCM group had markedly greater septal thickness/Z-scores than both DCM and controls (all pairwise *p* < 0.01), whereas DCM and controls did not differ. Left ventricle end-diastolic diameter differed among groups (*p* < 0.01), following HCM < Control < DCM (*p* <0.01). LVEDD Z-score was also higher in DCM than in HCM and controls (both *p* < 0.01), with no difference between HCM and controls.

In the A4C view, LAVi was significantly higher in both cardiomyopathy groups compared with controls (*p* < 0.05 for both comparisons). Correspondingly, LA EF A4C was markedly reduced in cardiomyopathy patients, averaging 54 ± 16.81% in DCM and 63.57 ± 12.24% in HCM, compared to 72.08 ± 7.32% in the control group. Full test statistics and summary measures by group are provided in Table 2.

### 3.4. Left Atrial Phasic Function

Significant intergroup differences were observed for LASr (*p* < 0.01). Patients with DCM exhibited markedly reduced LASr compared with both HCM (21.1± 11.8% vs. 34.3 ± 12.7%) and controls (21.1± 11.81% vs. 44.0 ± 11.4%), while HCM values were also lower than those in the control group (all pairwise *p* <0.05).

Conduit strain also differed significantly among groups, reflecting reduced passive emptying in both cardiomyopathy cohorts. Compared with controls (−32.7 ± 9.0%), LAScd was less negative in DCM (−15.8 ± 7.0%) and HCM (−20.8 ± 14.3%), both pairwise *p* < 0.01, with no difference between the two cardiomyopathy groups (*p* = 0.179).

Contractile strain was significantly attenuated in DCM (−5.0 ± 5.9%) compared with both HCM (−11.5 ± 6.8%) and controls (−10.5 ± 5.0%), with *p* < 0.01, whereas HCM and Control groups showed comparable values (*p* = 0.833).

Left ventricular global longitudinal strain was also significantly impaired across groups (*p* < 0.01). Both DCM and HCM demonstrated reduced GLS compared with controls (*p* < 0.01), while the DCM–HCM difference did not reach statistical significance (adjusted *p* = 0.262).

**Figure 2:**
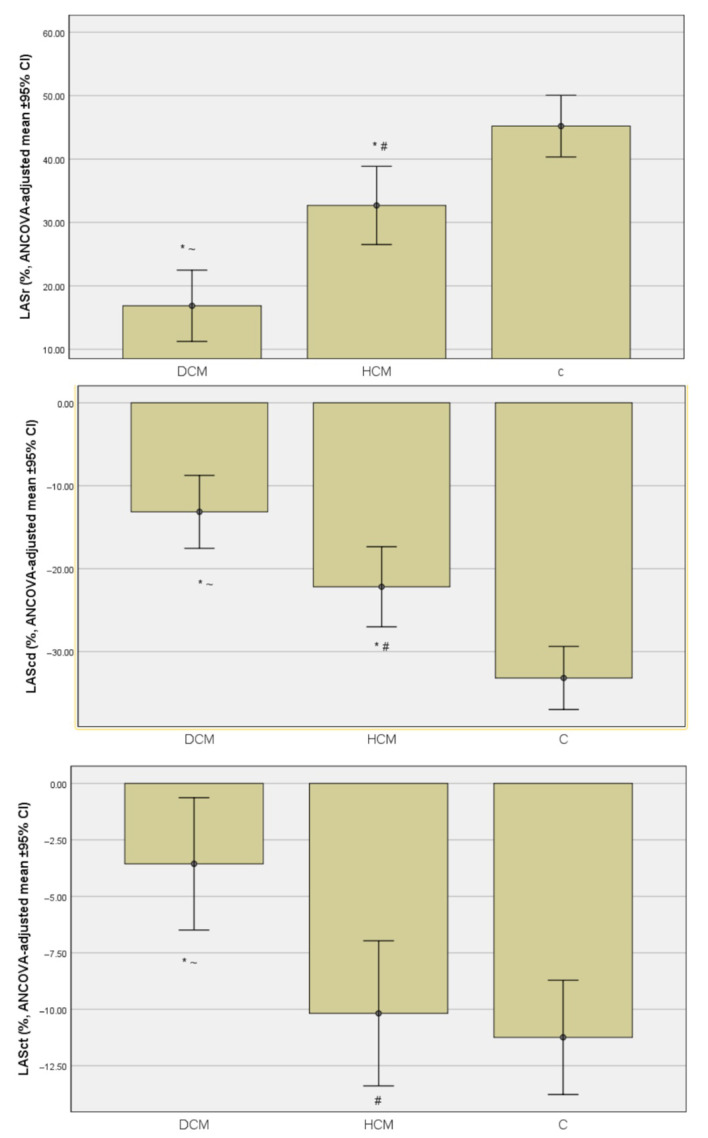
Comparison of LAS components among children with DCM, HCM, and healthy controls, adjusted for age, BSA, HR, and sBP percentile using analysis of covariance (ANCOVA). From the top, the first diagram shows left atrial reservoir strain (LASr); the second diagram, conduit strain (LAScd); and the third diagram, contractile strain (LASct). Bars represent ANCOVA-adjusted means with 95% confidence intervals. Overall group differences were significant for all parameters (*p* < 0.001 for LASr and LAScd; *p* = 0.001 for LASct). Post hoc Bonferroni comparisons: * *p* < 0.05 compared to control; # *p* < 0.05 compared to DCM; ~ *p* < 0.05 compared to HCM.

After adjustment for age, sBP percentile, BSA, HR, and BMI-z, these differences remained significant (*p* < 0.01) (Figure 2). All comparisons are shown in Table 3.

### 3.5. Correlations Between Cardiomyopathy Cohorts

In DCM, LASr correlated inversely with LAVi, z-log NT-proBNP, E/E′, and LVEDD z and correlated positively with e′ and LVEF (all *p* < 0.05), while LAScd and LASct were attenuated with increasing LAVi and markers of filling pressure (Table 4).

In HCM, LASr correlated inversely with IVSd z-score and z-log NT-proBNP, whereas LAScd worsened with both z-log NT-proBNP and IVSd z-score, but improved with higher E/A; other associations were non-significant.

### 3.6. Multivariate Linear Regression

In the DCM subgroup, higher LAVi and higher E/E′ were independently associated with lower LASr after adjustment for age and BSA (LAVi: β = −0.62; E/E′: β = −0.41, both *p* <0.05), whereas LVEF was not independently related to LASr (β = 0.18, *p*= 0.41).

In the HCM subgroup, IVSd z-score was the primary determinant of reduced LASr (β = −0.55, *p* < 0.01). Higher z-log NT-proBNP also showed an independent inverse association with LASr (β = −0.44, *p* <0.05). E/A ratio exhibited a borderline positive trend, while age and BSA were not significant in any model. All data were shown in Table 5.

### 3.7. Predictive Value of Left Atrial Strain Components for Functional Class Severity

**Figure 3:**
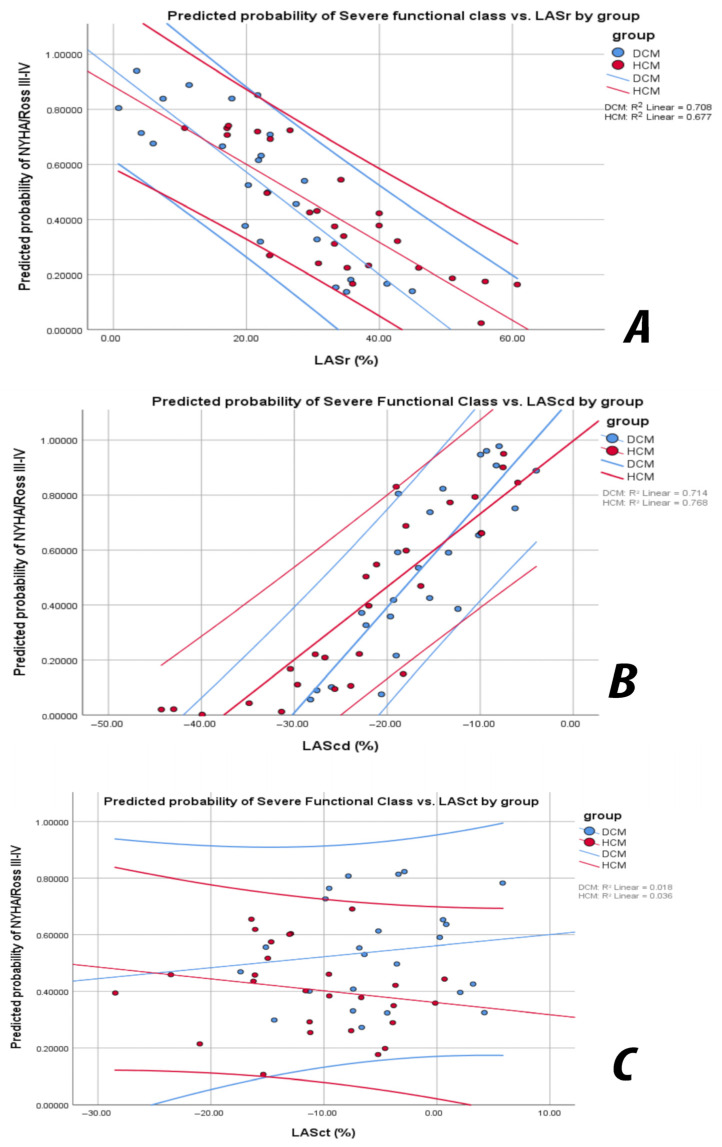
(**A**) Relationship between LASr and predicted probability of severe NYHA/Ross class (III–IV) in DCM and HCM groups. The downward slope indicates that lower LASr values are associated with an increased likelihood of functional limitation. The association was similar across both cardiomyopathy types. (**B**) Relationship between LAScd and predicted probability of severe NYHA/Ross class (III–IV). The downward-sloping trend illustrates that impaired conduit function (less negative strain) is associated with worse clinical status. (**C**) Relationship between LASct and predicted probability of severe NYHA/Ross class (III–IV). The weak and nonsignificant trend indicates that the atrial contraction phase contributes minimally to functional class prediction compared with reservoir and conduit components.

Binary logistic regression analysis, including age, BSA, HR, and cardiomyopathy type, showed that LASr was independently associated with severe NYHA/Ross class (χ^2^ = 13.55, *p* < 0.01; Nagelkerke R^2^ = 0.31). Each 1% decrease in LASr increased the odds of severe NYHA/Ross by 7% (OR = 0.93, 95% CI 0.88–0.99, *p* < 0.05). No significant effect was observed for age, BSA, HR, or cardiomyopathy subtype (Figure 3).

In the same multivariable model, LAScd was an even stronger and independent predictor of severe NYHA/Ross class (χ^2^ = 24.12, *p* < 0.01, Nagelkerke R^2^ = 0.50): each 1% reduction increased the probability of severe class by 21% (OR = 1.21, 95% CI 1.08–1.37, *p* < 0.01). In contrast, LASct was not significantly associated with NYHA/Ross class *(p* = 0.61).

These findings suggest that the reservoir and conduit phases of atrial deformation, rather than the contraction phase, are the primary determinants of symptomatic status in paediatric cardiomyopathy. To ensure robustness given the limited number of NYHA/Ross III-IV events, we performed a sensitivity analysis using Firth penalised logistic regression. This analysis confirmed the direction and significance of the associations identified in the standard logistic regression models, with LASr and LAScd remaining independently related to severe functional class.

## 4. Discussion

In this paediatric cohort, LA deformation parameters demonstrated distinct patterns across cardiomyopathy phenotypes. We observed a stepwise reduction in LASr, most pronounced in DCM and to a lesser extent in HCM. Similarly, LAScd was less negative in both cardiomyopathies, indicating reduced passive LV filling. Conversely, LASct was relatively preserved in HCM but markedly reduced in DCM. These differences remained significant after multivariable adjustment and may therefore provide phenotype-specific information on diastolic impairment. Left ventricular GLS, although reduced in both cardiomyopathy groups, did not differentiate between DCM and HCM. In paediatric DCM, LV GLS is a well-established sensitive marker of systolic dysfunction and clinical severity [28]. However, in our cohort, it did not clearly differentiate between HCM and DCM, suggesting that atrial strain parameters may offer complementary value in paediatric cardiomyopathy.

The association between LASr and LAScd and functional class provides additional clinical insight. Binary logistic regression analysis confirmed that both LASr and LAScd were strong, independent predictors of functional status, even after adjusting for body size and haemodynamic factors. In contrast, LASct did not independently predict functional class. These findings indicate that the reservoir and conduit phases, which reflect global atrial deformation and passive ventricular filling, may hold greater clinical relevance than contractile strain. Comparable findings have been reported in the adult population, where abnormal LAS was closely associated with higher NYHA class, even in the absence of LA enlargement [29,30]. Similarly, Raafs et al. demonstrated in adults with DCM that LASr and especially LAScd derived from cardiac MRI were superior to LV GLS, LV EF, and LAVi in predicting outcomes, suggesting that LAScd represents an early prognostic indicator of disease severity [4].

Given the cross-sectional design of our study, causal or mechanistic interpretations cannot be inferred. However, the patterns of association observed in our cohort are consistent with the existing paediatric and adult literature. In DCM, LASr showed strong correlations with LAVi and E/E`. In HCM, LAS parameters correlated instead with septal hypertrophy and NT-proBNP. This pattern is consistent with published paediatric HCM cohorts—where LASr and LAScd are decreased despite only modest LA volumetric changes—and with multicentre paediatric cardiomyopathy series emphasising the added value of LA mechanics over traditional Doppler indices [3,31,32]. These associations do not establish causal pathways, but they might indicate that the determinants of atrial deformation may differ between cardiomyopathy phenotypes.

Interestingly, while adult diastolic function algorithms define diastolic dysfunction when ≥2 of 4 ASE/EACVI criteria are abnormal (annular e′, E/e′, TR velocity, and LAVi >34 mL/m^2^, in our cohort, only a minority of patients crossed the adult LAVi abnormality threshold [10]. Furthermore, although an adult cut-off of E/E` ratio above 14 is used to indicate elevated LV filling pressure, most of our patients were below this threshold [10]. Despite significantly higher E/E` values in HCM than in controls, the substantial overlap with normal values supports prior paediatric studies that E/E` and LAVi alone have limited diagnostic value in children [13]. Although normative paediatric TDI values have been reported, consensus thresholds for defining elevated LV filling pressure remain undefined in paediatric populations [33]. This supports the idea that adult-based diastolic criteria have reduced sensitivity in paediatric patients and underscores the added value of LA strain measurements as supplementary markers.

Our data align with previously published paediatric studies while offering added comparative context. Jhaveri et al. evaluated LAS predominantly in paediatric HCM [3]. Dragulescu et al. explored diastolic dysfunction, conventional echocardiographic parameters in broader cardiomyopathy cohorts [13]. Sabatino et al. found that only children with restrictive cardiomyopathy exceeded the adult LAVi abnormality threshold, despite LAS reduction [32]. Similarly, in our DCM subgroup, LASr correlated inversely with LAVi, and LAScd showed a positive association. In contrast, in the HCM group, the associations between LAVi and all LAS parameters were non-significant, suggesting that in the hypertrophic disease, atrial function may be influenced by diastolic stiffness and impaired relaxation. Comparable trends were reported by Guan et al. in adults, where LASr and LAScd declined with worsening diastolic grades despite unchanged LAV in early disease [34]. Unlike previous paediatric studies, our work directly compares DCM, HCM, and healthy controls within a single cohort using consistent acquisition and analysis protocols. The inclusion of comprehensive LAS phasic components, integration with functional class, and NT-proBNP adjusted for age- and body size-related confounding provide additional methodological strength.

Regarding conventional echocardiographic parameters, in our cohort, transmitral inflow followed the U-shaped relation between E/A and LV filling pressure: the HCM cohort showed a higher A-wave with a lower E/A ratio, consistent with impaired relaxation and increased stiffness; the DCM cohort exhibited similar E wave values to controls, with intermediate E/A, compatible with pseudonormal filling [15]. This pattern, especially the shift in HCM driven by increased atrial booster pumping, may highlight the added sensitivity of LA strain parameters to detect diastolic dysfunction.

Overall, our results support the potential value of LA strain as an additional non-invasive marker of diastolic dysfunction and functional status in paediatric cardiomyopathy. Incorporating LASr and LAScd into echocardiographic assessment may enhance risk stratification and early identification of subclinical dysfunction, especially in patients with inconclusive conventional parameters. However, future longitudinal studies in larger, multicentre paediatric populations will be essential to determine whether LAS parameters carry incremental prognosis value or can guide clinical decision-making.

## 5. Limitations

The most important limitation of this study is its single-centre design and a relatively small sample size, which may reduce the statistical power for subgroup comparisons and limit the generalisability of the findings. Although all eligible patients were consecutively recruited during the study period, referral bias due to a national tertiary centre, particularly one that is specialised in advanced HF and transplantation, may influence the disease spectrum and severity represented in our cohort. Nevertheless, this institution also functions as the primary diagnostic and follow-up centre for a wide geographical region, ensuring a clinically diverse and wide paediatric cardiomyopathy population. The cross-sectional design of the study precludes causal inference and limits the ability to evaluate longitudinal changes or prognostic implications. In addition, potential confounding by loading conditions and medical therapy cannot be entirely excluded, as these factors may transiently affect LAS measurements. The wide age range of the cohort patients also represents a methodological limitation; however, we minimised age-related and size-related confounding by adjusting the analysis for age, body size-adjusted z-score, sBP percentiles and HR, which serve as surrogates for instantaneous loading conditions. Including a broad age range reflects real-world paediatric cardiomyopathy cohorts. Also, medication use may influence loading conditions and atrial deformations; however, due to the modest sample size and the large number of therapeutic combinations, these variables could not be incorporated into the multivariable models without risking overfitting. Therefore, residual confounding related to the medical therapy cannot be entirely excluded.

Another limitation lies in the absence of vendor-specific validation for LA strain analysis. Inter-vendor variability has been reported, primarily due to differences in the STE algorithms and post-processing methods. However, comparative studies that evaluated measurements obtained from different analysis platforms have shown that such variability exerts only a minor influence on the strength of associations between LAS and clinical or functional parameters [6,15,35]. This study did not include a formal assessment of STE feasibility and reproducibility, such as intra- and inter-observer variability. Despite this, prior investigations have reported the high feasibility and reproducibility of STE-derived LA strain measurements [15,35].

## 6. Conclusions

In this cross-sectional cohort of children with cardiomyopathy, LAS parameters demonstrated more pronounced differences between disease phenotypes than conventional echocardiographic parameters, reflecting distinct patterns of atrial dysfunction. Incorporating LA mechanics alongside conventional Doppler, LAVi (as z-scores), and biomarkers may enhance the characterisation of diastolic impairment and improve risk stratification approaches in paediatric clinical practice.

## Figures and Tables

**Table 1:** Baseline demographic, clinical, and treatment characteristics of the study population.

Variable	DCM (No. 29)	HCM (No. 29)	Control (No. 26)
Gender			
Male	72.4% (no. 21)	62.03% (no. 18)	50% (no. 13)
Female	27.6% (no. 8)	37.9% (no. 11)	50% (no. 13)
Age (yr)	14 [3.5–16]	13.83 [9.72–15.07]	15.44 [11.58–16.7]
Weight (kg)	41.57 ± 25.41	45.67 ± 22.47	53.54 ± 16.09
Weight z-score	0.01 ± 1.08	0.47± 0.82	−0.2 ± 1.64
Height (cm)	164 [101–173]	157 [132.5–168]	166 [154.3–171.3]
Height z-score	0.64 ± 1.05	0.61 ± 1.005	−0.07 ± 1.43
BMI (kg/m^2^)	18.2 ± 4.46	19 ± 4.18	20.28 ± 2.92
BMI z-score	−0.61 ± 1.62	0.2 ± 0.86	−0.21 ± 1.37
HR (bpm)	87.52± 17.03	79 ± 13.57	82.81 ± 18.69
sBP (mmHg)	102.9± 11.72	108.1± 9.66	105.9 ± 15.93
sBP percentile	59 [21–73]	48.5 [27.7–66.5]	62 [22–78.5]
BSA (m^2^)	1.56 [0.7–1.73]	1.55 [1.43–1.7]	1.44 [0.96–1.72]
NYHA/ROSS			
I	17.24% (n = 5)	24.13 (n = 7)	–
II	27.58% (n = 8)	41.37 (n = 12)	–
III	34.4% (n = 10)	34.48 (n = 10)	–
IV	20.69% (n = 6)	0	–
Arrythmias			
Ventricular premature beats	55.17% (n = 16)	31.03% (n = 9)	–
Ventricular tachycardia	27.28% (n = 8)	6.89% (n = 2)	–
Atrial arrhythmia	10.34% (n = 3)	6.89% (n = 2)	–
Treatment			
Beta-blocker	86.2% (n = 25)	82.75% (n = 24)	–
Calcium-blocker	–	3.44% (n = 1)	–
Diuretic	93.1% (n = 27) ~	3.44% (n = 1)	
ACEis	96.55% (n = 28) ~	–	–
Inotrop	34.48% (n = 10) ~	–	–
z-log NT-proBNP (log pg/mL)	3.28 ± 2.12 *~	1.99 ±1.99 *	0.31 ± 0.64

Continuous variables are expressed as the mean ± standard deviation (SD) or the median [interquartile range], depending on the data distribution. Categorical variables are presented as absolute frequencies and percentages. Omnibus comparisons were conducted using Kruskal–Wallis or ANOVA tests for continuous variables and χ^2^ test or Fisher’s exact test for categorical variables. Post hoc pairwise comparisons (Dunn–Holm or Tukey) were applied only for variables with significant group difference (* *p* < 0.01 compared to control; ~ *p* < 0.01 compared to HCM). Abbreviations: yr—years; cm—centimetres; BMI—body mass index; sBP—systolic blood pressure; BSA—body surface area; NYHA—New York Heart Association functional class; ACEis—angiotensin-converting enzyme inhibitors; z-log NT-proBNP—zlog transformation of N-terminal pro-B-type natriuretic peptide.

**Table 2:** Conventional echocardiography parameters among DCM, HCM, and control groups.

Variable	DCM (No. 29)	HCM (No. 29)	Control (No. 26)
E wave (cm/sec)	98.5 [82–110.8]	95.8 [78.5–126.5]	100 [80–122]
A wave (cm/sec)	64 [48–71]	73.6 [57.18–102.8] *	57 [48–64] ~
E/A	1.54 ± 0.4	1.37 ± 0.4 *	1.75 ± 0.35 ~
S lat (cm/sec)	7.45 ± 2.14	7.89 ± 1.94	11.08 ± 2.62
E` (cm/sec)	11.3 [8.92–18.85] *~	8.68 [4.58–21.1] *#	20.7 [17.25–23.69] ~#
A` (cm/sec)	6 [4.84–6.99]	7 [5.67–8.9]	8 [7.14–10.7]
E/e`	7.6 [5.69–11.91] *	11.68 [7.48–14.43] *	5.14 [4.35–6.86] ~#
E`/A`	2.19 ± 0.82	1.33 [0.74–1.94]	2.41 ± 0.68
MAPSE (cm)	1.02 ± 0.32	1.44 ± 0.29	1.73 ± 0.26
IVSs (cm)	1.03 ± 0.28 ~	2.44 ± 0.93 *#	1.28 ± 0.23 ~
IVSs (z score)	−0.38 ± 1.52~	9.59 ± 6.79 *#	0.48 ± 1.3 ~
IVSd (cm)	0.86 ± 0.3~	2.05 ± 1.1 *#	0.9 ± 0.21 ~
IVSd (z score)	0.57 ± 1.7 ~	9.36 ± 5.78 *#	0.12 ± 1.5 ~
LVEDD (cm)	5.57 ± 1.46 *	3.34 ± 0.77 *	4.33 ± 0.62 ~#
LVEDD-z score	4.29 [2.43–5.38] ~*	−3.8 [−4.9–−1.18] *	−0.87 ± 1.5 #
LVESD (cm)	4.43 ± 1.59	1.85 ± 0.73	2.5 ± 0.39
LVESD z score	6.14 [2.95–7.65]	−3.5 [−4.98–−1.8]	−1.26 [−2.2–−0.46]
LV EF (%)	42.59 ± 17.9 ~*	76.68 ± 10.5 #	72.4 ± 4.86 #
LV SF (%)	22.79 ± 9.89 ~*	45.54 ± 9.72 #	41.2 ± 4.27 #
LAVi A4C (mL/m^2^)	31.18 [21.24–43.59] *	27.07 [17.24–36.09] *	16.9 [14.81–19.95] ~#
LA EF A4C (%)	54 ± 16.81	63.57 ± 12.24	72.08 ± 7.32

Continuous variables are expressed as mean ± standard deviation (SD) or median [interquartile range], depending on distribution. *p*-values represent omnibus comparisons among groups (Kruskal–Wallis or ANOVA, as appropriate). Post hoc analyses were performed using Dunn–Holm or Tukey correction for multiple testing. Abbreviations: Cm—centimetres; MAPSE—mitral annular plane systolic excursion; IVS—interventricular septum (d—diastole; s—systole); IVS z-score—intraventricular septal thickness in systole/diastole standardised for age and body size; LVEDD—left ventricle end diastolic dimension; LVEDD z-score—left ventricle end diastolic dimensions standardised for age and body size; LVESD—left ventricle systolic dimension; LVESD z-score—left ventricle end systolic dimension standardised for age and body size; LV EF—left ventricle ejection fraction; LV SF—left ventricle shortening fraction; LAVi—left atrial volume indexed to body surface area; mL—millilitres; LA EF—left atrial ejection fraction; A4C—apical four-chamber view. * *p* < 0.01 compared to control; # *p* < 0.01 compared to DCM; ~ *p* < 0.01 compared to HCM.

**Table 3:** Speckle-tracking echocardiography–derived LAS and LV global longitudinal strain (GLS) parameters in DCM, HCM, and control groups.

Variable	DCM (No. 29)	HCM (No. 29)	Control (No. 26)
LASr ED (%)	21.13 ± 11.81 ~*	34.39 ± 12.66 *#	44 ± 11.43 ~#
LAScd ED (%)	−15.76 ± 6.991 *	−20.75 ± 14.32 *	−32.7 ± 8.95 ~#
LASct ED (%)	−5.02 ± 5.89 ~*	−11.46± 6.78 #	−10.53 ± 4.96 #
LV GLS (%)	−11.71 ± 6.51	−14.98 ± 4.06	−19.93 ± 3.24

Data are presented as mean ± standard deviation (SD). *p*-values represent omnibus comparisons across the three study groups (one-way ANOVA for normally distributed variables or Kruskal–Wallis test otherwise), followed by Bonferroni- or Dunn–Holm-adjusted pairwise comparisons as appropriate. Abbreviations: LASr (ED)—left atrial reservoir strain at end-diastole; LAScd (ED)—left atrial conduit strain at end-diastole; LASct (ED)—left atrial contractile strain at end-diastole; LV GLS—left ventricular global longitudinal strain. * *p* < 0.05 compared to control; # *p* < 0.05 compared to DCM; ~ *p* < 0.05 compared to HCM.

**Table 4:** Correlation coefficients between LAS components and echocardiographic or biochemical parameters in the DCM and HCM subgroups.

Group	DCM			HCM		
Parameter (R)	LASr	LAScd	LASct	LASr	LAScd	LASct
z-log NT-proBNP	**−0.62**	**0.6**	0.34	**−0.39**	**0.61**	0.11
E (cm/sec)	−0.09	0.03	0.23	0.09	−0.13	−0.06
E/A	−0.2	0.38	**0.45**	0.11	**−0.54**	0.08
E` (cm/sec)	0.43	**−0.48**	−0.14	0.35	−0.27	−0.31
E/E`	**−0.48**	**0.55**	0.18	−0.21	0.17	0.13
IVSd z-score	0.05	0.1	0.02	**−0.49**	**0.58**	0.1
LVEDD z-score	**−0.42**	0.26	0.08	**0.36**	**−0.37**	−0.12
LV EF (%)	**0.53**	**−0.38**	−0.21	−0.14	0.25	−0.06
LAVi (mL/m^2^)	**−0.66**	**0.73**	**0.47**	−0.18	0.22	−0.04

Data represent Pearson or Spearman correlation coefficients, depending on data distribution. Statistically significant correlations (*p* < 0.05) are highlighted in bold. Negative correlations indicate inverse relationships between parameters and components of left atrial strain. Abbreviations: LASr—left atrial reservoir strain; LAScd—left atrial conduit strain; LASct—left atrial contractile strain; z-log NT-proBNP—standardised log-transformed N-terminal pro-B-type natriuretic peptide); IVSd z-score—interventricular septal diastolic thickness standardised for age and body size; LVEDD z-score—left ventricular end-diastolic diameter standardised for age and body size; LV EF—left ventricular ejection fraction; LAVi—left atrial volume indexed to body surface area;.

**Table 5:** Multivariable linear regression analysis for determinants of left atrial reservoir strain (LASr) in DCM and HCM subgroups.

Group/Predictor	Regression Coefficient b (95% CI)	*p*-Value	Adjusted R^2^
**DCM**			
LAVi (mL/m^2^)	−0.39 (−0.68 to −0.11)	0.01	0.28
E/E′ (–)	−1.11 (−2.14 to −0.08)	0.04	0.16
LV EF (%)	+0.12 (−0.18 to +0.42)	0.41	0
**HCM**			
IVSd Z-score	−1.24 (−2.09 to −0.38)	0.007	0.23
z-log NT-proBNP	−2.77 (−5.19 to −0.35)	0.027	0.08
E/A ratio	+13.59 (−0.65 to +27.83)	0.06	0.14

Separate regression models were constructed for each cardiomyopathy group, with LASr (%) entered as the dependent variable. Independent variables were selected based on significant or borderline univariate correlations and included echocardiographic and biochemical parameters reflecting ventricular function and filling pressure. All models were adjusted for age and body surface area (BSA). Abbreviations: LAVi—left atrial volume indexed to body surface area; LV EF—left ventricular ejection fraction; IVSd Z-score—interventricular septal thickness in diastole, standardised for age and body size; z-log NT-proBNP—standardised log-transformed N-terminal pro–B-type natriuretic peptide.

## Data Availability

Data can be made available upon request.

## Abbreviations

The following abbreviations are used in this manuscript:

LA	Left atrium
LAS	Left atrium strain
DCM	Dilated cardiomyopathy
HCM	Hypertrophic cardiomyopathy
LV	Left ventricle
MRI	Magnetic resonance imaging
LASr	Left atrial reservoir strain
LAScd	Left atrial conduit strain
LASct	Left atrial contractile strain
HR	Heart rate
BP	Blood pressure
BSA	Body surface area
ASE	American Society of Echocardiography
TDI	Tissue Doppler-derived indices
STE	Speckle-tracking echocardiography
A4C	Apical four-chamber
A3C	Apical three-chamber
A2C	Apical two-chamber
HF	Heart failure
ED	End-diastole
LVEDD	Left ventricle end-diastolic diameter
SD	Standard deviation
BMI	Body mass index
ACEi	Angiotensin-converting enzyme inhibitors
NYHA	New York Heart Association
NT-proBNP	N-terminal pro-B-type natriuretic peptide
MAPSE	Mitral annular plane systolic excursion
IVS	Interventricular septum
LVEDD	Left ventricle end-diastolic dimension
LVESD	Left ventricle end-systolic dimension
LV EF	Left ventricular ejection fraction
LV SF	Left ventricle shortening fraction
LAVi	Left atrial volume indexed
LA EF	Left atrial ejection fraction
IVS	Interventricular septal thickness
